# Review on Natural Coumarin Lead Compounds for Their Pharmacological Activity

**DOI:** 10.1155/2013/963248

**Published:** 2013-03-24

**Authors:** K. N. Venugopala, V. Rashmi, B. Odhav

**Affiliations:** ^1^Department of Biotechnology and Food Technology, Durban University of Technology, Durban 4001, South Africa; ^2^Department of Public Health Medicine, University of KwaZulu-Natal, Howard College Campus, Durban 4001, South Africa

## Abstract

Coumarin (2*H*-1-benzopyran-2-one) is a plant-derived natural product known for its pharmacological properties such as anti-inflammatory, anticoagulant, antibacterial, antifungal, antiviral, anticancer, antihypertensive, antitubercular, anticonvulsant, antiadipogenic, antihyperglycemic, antioxidant, and neuroprotective properties. Dietary exposure to benzopyrones is significant as these compounds are found in vegetables, fruits, seeds, nuts, coffee, tea, and wine. In view of the established low toxicity, relative cheapness, presence in the diet, and occurrence in various herbal remedies of coumarins, it appears prudent to evaluate their properties and applications further.

## 1. Introduction

**Scheme 1:**
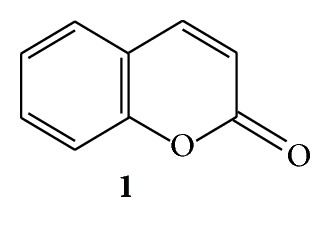


Coumarins (2*H*-1-benzopyran-2-one) (**1**) consist of a large class of phenolic substances found in plants and are made of fused benzene and *α*-pyrone rings [1]. More than 1300 coumarins have been identified as secondary metabolites from plants, bacteria, and fungi [2]. The prototypical compound is known as 1,2-benzopyrone or, less commonly, as *o*-hydroxycinnamic acid and lactone, and it has been well studied. Coumarins were initially found in tonka bean (*Dipteryx odorata* Wild) and are reported in about 150 different species distributed over nearly 30 different families, of which a few important ones are Rutaceae, Umbelliferae, Clusiaceae, Guttiferae, Caprifoliaceae, Oleaceae, Nyctaginaceae, and Apiaceae. (See Scheme 1.)

Although distributed throughout all parts of the plant, the coumarins occur at the highest levels in the fruits (Bael fruits (*Aegle marmelos*) [3], *Tetrapleura tetraptera* TAUB (Mimosaceae) [4], bilberry, and cloudberry), seeds (tonka beans) (*Calophyllum cerasiferum* Vesque and *Calophyllum inophyllum* Linn) [5] followed by the roots (*Ferulago campestris*) [6], leaves (*Murraya paniculata*) [7], *Phellodendron amurense* var. *wilsonii* [8], and latex of the tropical rainforest tree *Calophyllum teysmannii* var. inophylloide [9] green tea and other foods such as chicory. They are also found at high levels in some essential oils such as cassia oil [10], cinnamon bark oil [11], and lavender oil [6]. Environmental conditions and seasonal changes could influence the incidence of coumarins in varied parts of the plant. The function of coumarins is far from clear, although suggestions include plant growth regulators, bacteriostats, fungistats, and even waste products [12].

Biosynthesis of coumarin is reviewed by Bourgaud et al. [11]. There are types of coumarins found in nature due to various permutations brought about by substitutions and conjugations; however, most of the pharmacological and biochemical studies have been done on coumarin itself and on its primary metabolite, 7-hydroxycoumarin in man [13]. Some of this earlier pharmacological work on coumarin has been reviewed [14], and other more comprehensive reviews [13, 15, 16] deal with the occurrence, chemistry, and biochemical properties of both simple and more complex natural coumarins. 

## 2. Classification of Coumarins

Natural coumarins are mainly classified into six types based on the chemical structure of the compounds (Table 1). The physicochemical properties and therapeutic applications of natural coumarins depend upon the pattern of substitution.

## 3. Coumarins and Pharmacological Activity

### 3.1. Coumarins for Anti-Inflammatory Activity

**Scheme 2:**
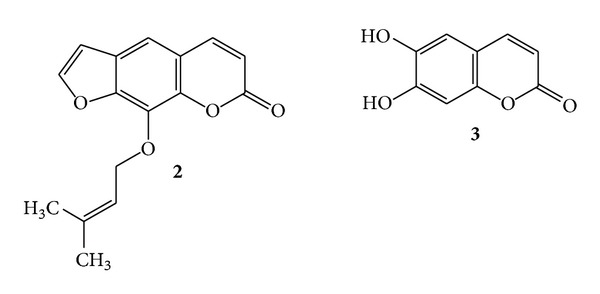


Coumarin (**1**) exhibits anti-inflammatory property and is used in the treatment of oedema. This removes protein and oedema fluid from injured tissue by stimulating phagocytosis, enzyme production, and thus proteolysis [17]. Another compound imperatorin (**2**) also shows anti-inflammatory activity in lipopolysaccharide-stimulated mouse macrophage (RAW264.7) *in vitro* and a carrageenan-induced mouse paw edema model *in vivo*. Imperatorin blocks the protein expression of inducible nitric oxide synthase and cyclooxygenase-2 in lipopolysaccharide-stimulated RAW264.7 [39]. Esculetin (**3**) was isolated from *Cichorium intybus *[40] and *Bougainvillea spectabilis *Wild (Nyctaginaceae) [41]. It exhibited anti-inflammatory activity in rat colitis induced by trinitrobenzenesulfonic acid [18, 42]. Esculetin (**3**) inhibits the cyclooxygenase and lipoxygenase enzymes, also of the neutrophil-dependent superoxide anion generation [43]. (See Scheme 2.)

### 3.2. Coumarins for Anticoagulant Activity

**Scheme 3:**
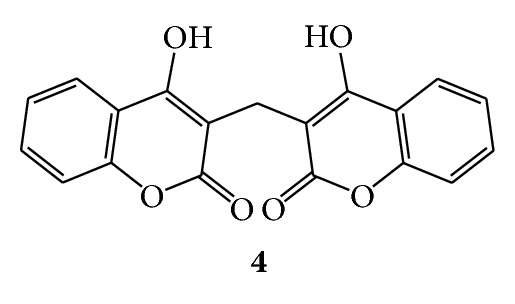


Dicoumarol (**4**) was found in sweet clover [1] and exhibited anticoagulant activity [38]. (See Scheme 3.)

**Figure 1:**
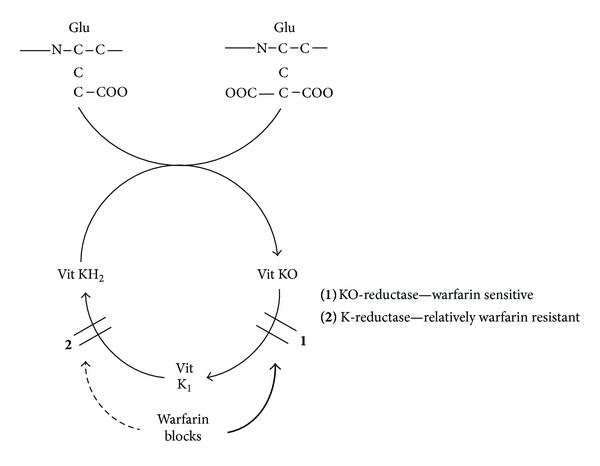
Coumarin analogue warfarin and vitamin K cycle.

Coumarins are vitamin K antagonists that produce their anticoagulant effect by interfering with the cyclic interconversion of vitamin K and its 2,3 epoxide (vitamin K epoxide) [44]. Vitamin K is a cofactor for the posttranslational carboxylation of glutamate residues to *γ*-carboxyglutamates on the *N*-terminal regions of vitamin K-dependent proteins (Figure 1) [45–50].

These coagulation factors (factors II, VII, IX, and X) require *γ*-carboxylation for their biological activity. Coumarins produce their anticoagulant effect by inhibiting vitamin K conversion cycle, thereby causing hepatic production of partially carboxylated and decarboxylated proteins with reduced procoagulant activity [51, 52]. In addition to their anticoagulant effect, vitamin K antagonists inhibit carboxylation of the regulatory anticoagulant proteins C and S and therefore have the potential to exert a procoagulant effect. In the presence of calcium ions, carboxylation causes a conformational change in coagulation proteins [53–55] that promotes binding to cofactors on phospholipid surfaces. The carboxylation reaction requires the reduced form of vitamin K (vitamin KH_2_), molecular oxygen, and carbon dioxide and is linked to the oxidation of vitamin KH_2_ to vitamin K epoxide. Vitamin K epoxide is then recycled to vitamin KH_2_ through two reductase steps. The first, which is sensitive to vitamin K antagonist [47, 49, 50], reduces vitamin K epoxide to vitamin K_1_ (the natural food form of vitamin K_1_), while the second, which is relatively insensitive to vitamin K antagonists, reduces vitamin K_1_ to vitamin KH_2_. Treatment with vitamin K antagonists leads to the depletion of vitamin KH_2_, thereby limiting the *γ*-carboxylation of vitamin K-dependent coagulant proteins. The effect of coumarins can be counteracted by vitamin K_1_ (either ingested in food or administered therapeutically) because the second reductase step is relatively insensitive to vitamin K antagonists (Figure 1). Patients treated with a large dose of vitamin K_1_ can also become warfarin resistant for up to a week because vitamin K_1_ accumulates in the liver and is available to the coumarin-insensitive reductase.

### 3.3. Coumarins for Antibacterial Activity

**Scheme 4:**
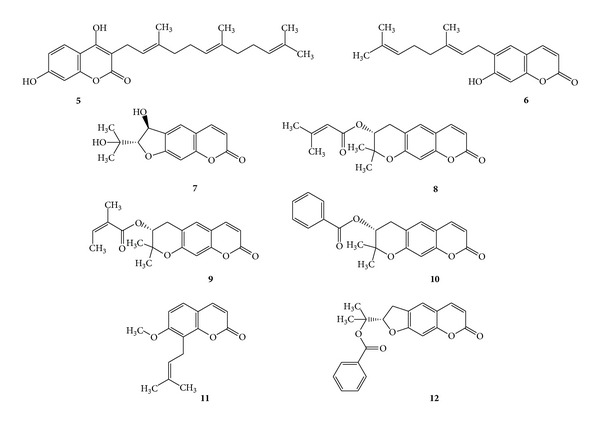


Coumarin (**1**) itself has a very low antibacterial activity, but compounds having long chain hydrocarbon substitutions such as ammoresinol (**5**) and ostruthin (**6**) show activity against a wide spectrum of Gram +ve bacteria such as *Bacillus megaterium*, *Micrococcus luteus*, *Micrococcus lysodeikticus*, and *Staphylococcus aureus* [19]. Another coumarin compound anthogenol (**7**) from green fruits of *Aegle marmelos* [3] shows activity against *Enterococcus*. Imperatorin (**2**), a furanocoumarin isolated from *Angelica dahurica* and *Angelica archangelica *(Umbelliferae) [56], shows activity against *Shigella dysenteriae* [57]. Grandivittin (**8**), agasyllin (**9**), aegelinol benzoate (**10**) and osthole (**11**) have been isolated from the roots of *Ferulago campestris* (Apiaceae) [32]. Felamidin (**12**) was also isolated from *Ferulago campestris* [6]. Aegelinol and agasyllin showed significant antibacterial activity against clinically isolated Gram-positive and Gram-negative bacterial strains such as *Staphylococcus aureus*, *Salmonella typhi*, *Enterobacter cloacae,* and *Enterobacter aerogenes*. Antibacterial activity was also found against *Helicobacter pylori* where a dose-dependent inhibition was shown between 5 and 25 mg/mL. (See Scheme 4.)

**Scheme 5:**
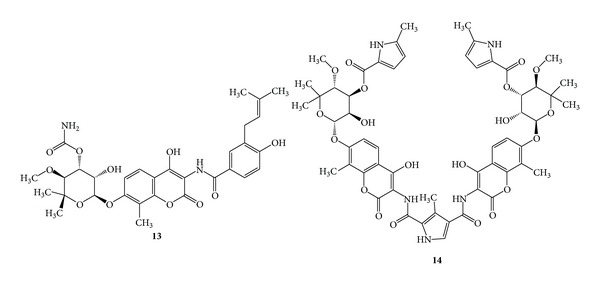


Many of the natural coumarins in existence have been isolated from higher plants; some of them have been discovered in microorganisms. The important coumarin members belonging to microbial sources are novobiocin, coumermycin, and chartreusin. Novobiocin (**13**) was isolated as fungal metabolite from *Streptomyces niveus* [58] and *Streptomyces spheroides* and has exhibited broad spectrum antibacterial activity against Gram-positive organisms such as *Corinebacterium diphtheria*, *Staphylococcus aureus*, *Streptomyces pneumoniae*, and *Streptomyces pyogenes* and Gram-negative organisms such as *Haemophillus influenzae*, *Neisseria meningitides,* and *Pasteurella* [21] and has shown DNA gyrase inhibition activity [22]. Coumermycin (**14**), that is, structurally similar to novobiocin is nearly 50 times more potent than novobiocin, against *Escherichia coli* and *Staphylococcus aureus*, but it produces a bacteriostatic action, and the organism developed resistance gradually. Coumermycin also inhibits the supercoiling of DNA catalyzed by *Escherichia coli* DNA gyrase [22]. (See Scheme 5.)

**Scheme 6:**
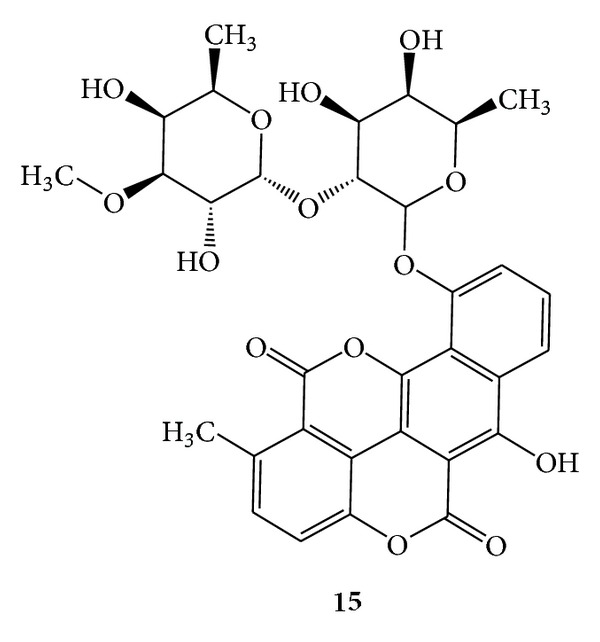


Chartreusin (**15**) was isolated from *Streptomyces chartreusis* and has an uncommon structure and was predominantly active against Gram-positive bacteria [38], but due to its toxicity, the compound has not been tried for therapeutic application. (See Scheme 6.)

### 3.4. Coumarins for Antifungal Activity

**Scheme 7:**
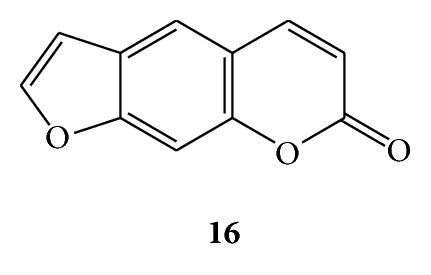


Osthole (**11**) is a bioactive coumarin derivative extracted from medicinal plants such as *Angelica pubescens *[59], *Cnidium monnieri *[60], and *Peucedanum ostruthium *[61]. Osthole exhibited wide spectrum of antifungal activity against important plant pathogens such as *Rhizoctonia solani*, *Phytophtora capsici*, *Botrytis cinerea*, *Sclerotinia sclerotiorum,* and *Fusarium graminearum *[20]. A number of coumarins have been tested for antifungal activity, and the three most effective ones are psoralen (**16**) [11], imperatorin (**2**), and ostruthin (**6**). (See Scheme 7.)

### 3.5. Coumarins for Antiviral Activity

**Scheme 8:**
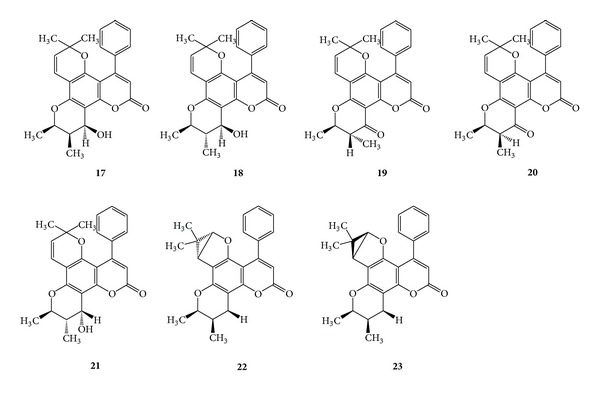


A large variety of natural products have been described as anti-HIV agents, and compounds having coumarin nucleus are among them. The inophyllums and calanolides represent novel HIV inhibitory coumarin derivatives. Inophyllum A (**17**), inophyllum B (**18**), inophyllum C (**19**), inophyllum E (**20**), inophyllum P (**21**), inophyllum G1 (**22**), and inophyllum G2 (**23**) were isolated from giant African snail, *Achatina fulica.* Inophyllums B and P (**18** and **21**) inhibited HIV reverse transcriptase (RT) with IC_50_ values of 38 and 130 nM, respectively, and both were active against HIV-1 in cell culture (IC_50_ of 1.4 and 1.6 *μ*M) [33]. (See Scheme 8.)

**Scheme 9:**
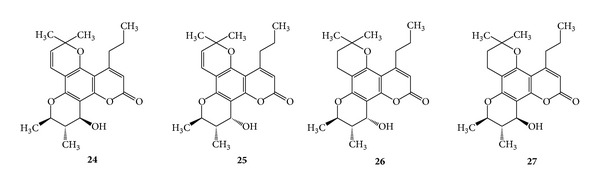


Two isomers, (+)-calanolide A (**24**) and (−)-calanolide B (**25**), have been isolated from the leaves of *Calophyllum lanigerum *(Clusiaceae). Calanolides A and B were completely protective against HIV-1 replication [34]. (+)-Calanolide A is a nonnucleoside RT inhibitor with potent activity against HIV-1. (−)-Calanolide B and (−)-dihydrocalanolide B (**26**) possess antiviral properties similar to those of (+)-calanolide A [35, 62]. Both (+)-calanolide A and (+)-dihydrocalanolide A (**27**) are stable at neutral pH and currently under development for the treatment of HIV infections. However, at a pH < 2.0 for 1 h, 73% of the (+)-calanolide A was converted to (+)-calanolide B while 83% of (+)-dihydrocalanolide A was converted to (+)-dihydrocalanolide B [35, 62]. Previously inophyllum A (**17**) and (−)-calanolide B (**25**) were isolated from the oil of seeds of *Calophyllum inophyllum *Linn and *Calophyllum cerasiferum *Vesque, respectively. Both of them belong to the family Clusiaceae and are known for potent HIV-1 RT inhibitors [5]. (See Scheme 9.)

**Scheme 10:**
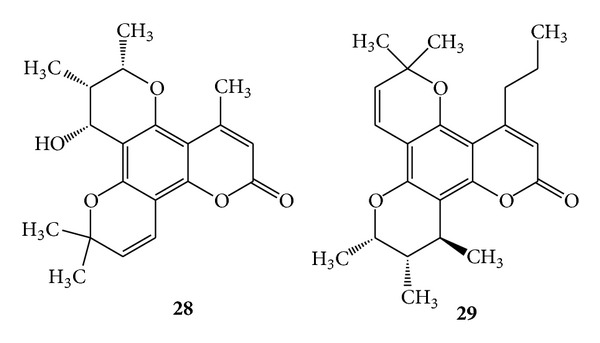


Pyranocoumarins such as pseudocordatolide C (**28**) and calanolide F (**29**) were isolated from extracts of *Calophyllum lanigerum* var. austrocoriaceum and *Calophyllum teysmannii* var. inophylloide (King) P. F. Stevens (Clusiaceae). Both the compounds exhibited anti-HIV activity [36]. Imperatorin (**2**) also inhibits either vesicular stomatitis virus pseudotyped or gp160-enveloped recombinant HIV-1 infection in several T-cell lines and in HeLa cells [63]. (See Scheme 10.)

### 3.6. Coumarins for Anticancer Activity

**Scheme 11:**
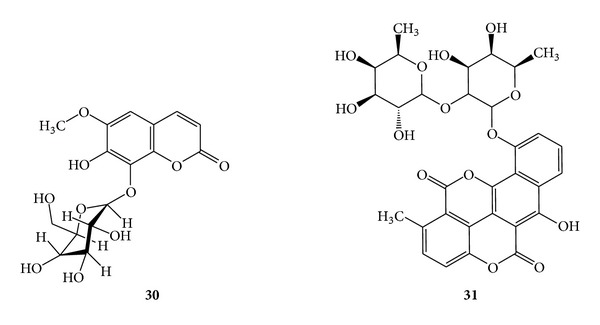


Imperatorin (**2**) exhibited anticancer effects [64]. Osthole (**11**) is effective in inhibiting the migration and invasion of breast cancer cells by wound healing and transwell assays. Luciferase and zymography assays revealed that osthole effectively inhibits matrix metalloproteinase-s promoter and enzyme activity, which might be one of the causes that lead to the inhibition of migration and invasion by osthole [65]. Esculetin (**3**) exhibited antitumor activities [66] and rescues cultured primary neurons from *N*-methyl-D-aspartate toxicity [67]. Protective effects of fraxin (**30**) against cytotoxicity induced by hydrogen peroxide were examined in human umbilical vein endothelial cells [24]. Most of the coumarins grandivittin (**8**), agasyllin (**9**), aegelinol benzoate (**10**), and osthole (**11**) from *Ferulago campestris* plant exhibited marginally cytotoxic activity against the A549 lung cancer cell line [6]. Chartreusin (**15**) was shown to exhibit antitumor properties against murine L1210, P388 leukemias, and B16 melanoma [23]. 3′′-Demethylchartreusin (**31**) is a novel antitumor antibiotic produced by *Streptomyces chartreusis* and it was a structural analogue of chartreusin containing the same aglycone of chartreusin, but different sugar moieties [38]. (See Scheme 11.)

Coumarin (**1**) which is isolated form cassia leaf oil exhibited cytotoxic activity [10].

### 3.7. Coumarins for Antihypertensive Activity

**Scheme 12:**
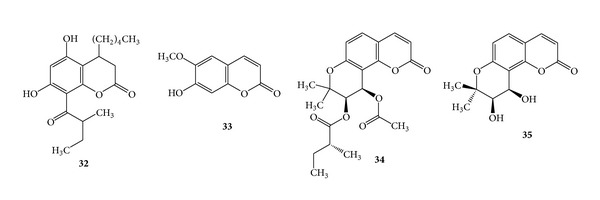


Dihydromammea C/OB (**32**) is a new coumarin that has been isolated from the seeds of the West African tree *Mammea africana* Sabine (Guttiferae) [68]. The molecular structure has been elucidated by single crystal X-ray method [69]. Antihypertensive effects of the methanol and dichloromethane extracts of stem bark from *Mammea africana* in N^*ω*^-nitro-L-arginine methyl ester induced hypertensive male albino Wistar rats weighing 250–300 g of 12–16-week old rats have been used in the studies [70]. Dichloromethane and methanol extracts of stem bark from *Mammea africana* exhibited a significant antihyperglycemic activity and improved the metabolic alterations in streptozotocin-induced male albino Wistar diabetic rats (3-month-olds, weighing 200–250 g) [71]. Vasodilatory effects of the coumarin are reported on cultured myocardial cells as well [72]. Scopoletin (**33**) was isolated form the fruits of *Tetrapleura tetraptera* TAUB (Mimosaceae) and it produces hypotension in laboratory animals *in vitro* and *in vivo* through its smooth muscle relaxant activity [4]. Visnadine (**34**), an active ingredient extracted from the fruit of *Ammi visnaga,* exhibited peripheral and coronary vasodilator activities and has been used for the treatment of angina pectoris [2]. Khellactone (**35**) was isolated from *Phlojodicarpus sibiricus* and it exhibited vasodilatory action [73]. (See Scheme 12.)

### 3.8. Coumarins for Antitubercular Activity

**Scheme 13:**
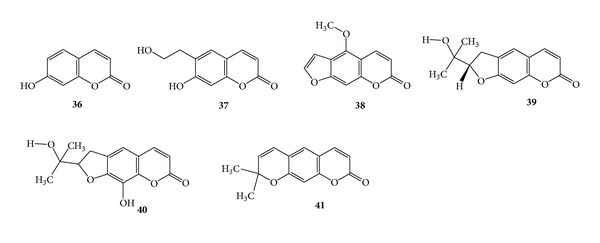


Umbelliferone (**36**) is found in many plants and obtained by the distillation of resins belonging to the natural order Umbelliferae [27]. Umbelliferone (**36**), phellodenol A (**37**), psoralen (**16**) and scopoletin (**33**), bergapten (**38**), (+)-(*S*)-marmesin (**39**), (+)-(*S*)-rutaretin (**40**), and xanthyletin (**41**) were isolated from the whole plants of *Fatoua pilosa*. The compounds scopoletin and umbelliferone are found to be active against *Mycobacterium tuberculosis* H_37_Rv with MIC values of 42 and 58.3 *μ*g/mL, respectively [25]. Compounds phellodenol A, (+)-(*S*)-marmesin and xanthyletin exhibited activity at 60 *μ*g/mL and the remaining compounds exhibited activity at more than 119 *μ*g/mL. Phellodenol A was also isolated from the leaves of *Phellodendron amurense* var. *wilsonii* [8]. (See Scheme 13.)

### 3.9. Coumarins for Anticonvulsant Activity

Imperatorin (**2**) showed anticonvulsant action in mice and the ED_50_ values ranged between 167 and 290 mg/kg. Acute neurotoxic effects in the chimney test revealed that the TD_50_ values for imperatorin ranged between 329 and 443 mg/kg [56]. Osthole (**11**) exhibited anticonvulsant action in mice and the ED_50_ values ranged between 253 and 639 mg/kg and the acute neurotoxic effects with the TD_50_ values ranged between 531 and 648 mg/kg [74]. 

### 3.10. Coumarins for Multiple Sclerosis

Osthole (**11**) could be a potential therapeutic agent for the treatment of multiple sclerosis [75]. 

### 3.11. Coumarins for Antiadipogenic Activity

**Scheme 14:**
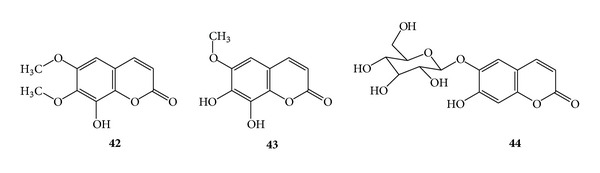


Fraxidin (**42**), [26] fraxetin (**43**), fraxin (**30**), esculetin (**3**), esculin (**44**), and scopoletin (**33**) have been isolated from the stem barks of *Fraxinus rhynchophylla *DENCE (Oleaceae). Esculetin (**3**) showed the most potent antiadipogenic activity against preadipocyte cell line, 3T3-L1 by *in vitro *assay system [27]. (See Scheme 14.)

### 3.12. Coumarins for Cytochrome P450 Inhibiting Activity

**Scheme 15:**
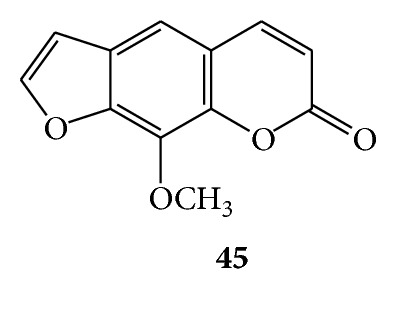


Methoxsalen (8-methoxypsoralen) (**45**) is found in the seeds of the *Ammi majus* (Umbelliferae) and exhibited potent mechanism-based microsomal P 450 inhibitor *in vitro* [76] and single-dose methoxsalen effects on human cytochrome P 450 2A6 activity [30]. (See Scheme 15.)

### 3.13. Coumarins for Antihyperglycemic Activity

Fraxidin (**42**) inhibited the formation of inducible nitric oxide synthase [77] and showed antihyperglycemic activity [78]. 

### 3.14. Coumarins for Antioxidant Activity

Fraxin (**30**) showed free radical scavenging effect at high concentration (0.5 mM) and cell protective effect against H_2_O_2_-mediated oxidative stress [24]. Esculetin (**3**) exhibited antioxidant property [79]. The antioxidant activity of the coumarins grandivittin (**8**), agasyllin (**9**), aegelinol benzoate (**10**), and osthol (**11**) was evaluated by their effects on human whole blood leukocytes and on isolated polymorphonucleated chemiluminescence [32]. Fraxin (**30**) and esculin (**44**) were characterized in stems and fruits of *Actinidia deliciosa* (kiwifruit) and *Actinidia chinensis *[80]. Fraxin (**30**) extracted from *Weigela florida* var. glabra leaves (Caprifoliaceae) protects cells from oxidative stress. 

### 3.15. Coumarins for Neuroprotective Activity

Esculetin (**3**) also exhibited neuroprotective effects on cerebral ischemia/reperfusion injury in a middle cerebral artery occlusion model in mice at 20 *μ*g/mL and was administered intracerebroventricularly at 30 min before ischemia [81].

### 3.16. Coumarins as Phytoalexins

**Scheme 16:**
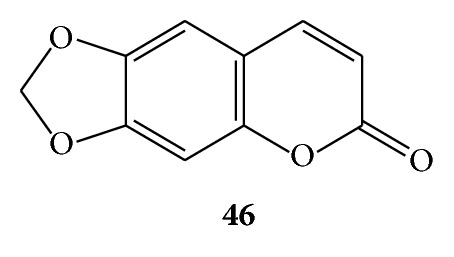


Phytoalexins are oxygenated coumarin derivatives and they are produced in plants in response to fungal infection, physical damage, chemical injury, or a pathogenic process. The common property of phytoallexins is to inhibit or destroy the invading agents such as bacteria, insects, and viruses. Ayapin (**46**) is one among them and structurally it is 6,7-methylenedioxycoumarin. Initially it was isolated from *Eupatorium ayapana* (Asteraceae) [4]. Later, ayapin (**46**) was isolated from a number of other plants such as *Helianthus annuus *[8], *Artemisia apiacea* [2], *Pterocaulon virgatum* [14], and *Pterocaulon polystachyum* [15]. (See Scheme 16.)

## 4. Identification of Coumarins from Different Sources and Their Structural Elucidation

**Scheme 17:**
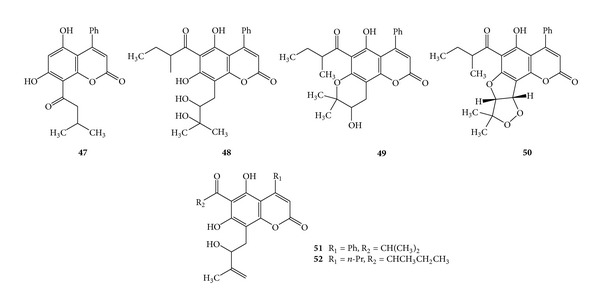


Coumarin compounds isodispar B (**47**), dispardiol B, (**48**), mammea A/AB cyclo E (**49**), mammea A/AB dioxalanocyclo F (**50**), disparinol D (**51**), and disparpropylinol B (**52**) have been isolated from the fruits and the stem bark of *Calophyllum dispar *(Clusiaceae) [37, 82, 83]. (See Scheme 17.)

Seed oil [5] and essential oils such as cinnamon bark oil [11] and lavender oil from roots (*Ferulago campestris*) [6], contain some amount of coumarin compound (**1**). 

**Scheme 18:**
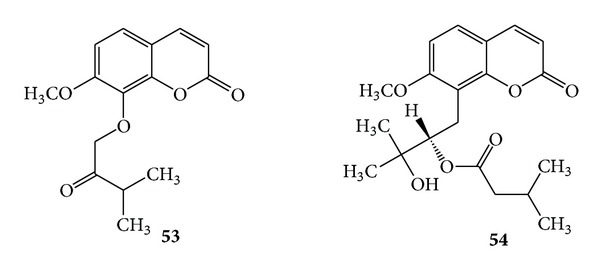


The main coumarin constituents found from the leaves of *Murraya paniculata* are 7-methoxy-8-(3-methyl-2-oxobutoxy)-2*H*-chromen-2-one (**53**) [7] and murrayatin (**54**). The latter was also found in the leaves of *Murraya exotica* [28]. (See Scheme 18).

**Scheme 19:**
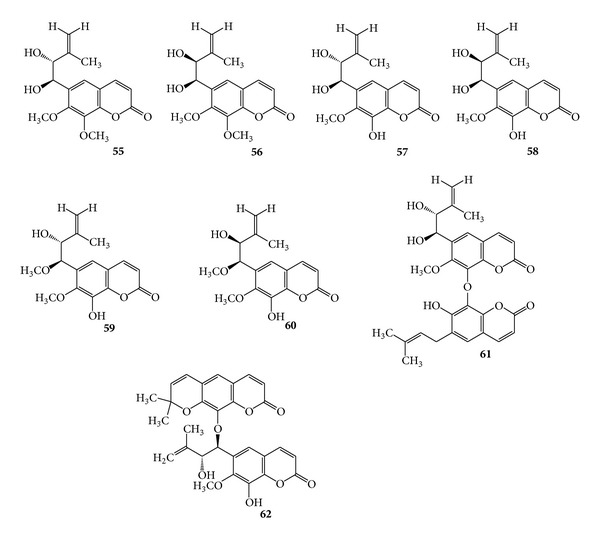


Prenylcoumarins (+)-fatouain A (**55**), (+)-fatouain A (**56**), (+)-fatouain C (**57**), (−)-fatouain D (**58**), (+)-fatouain E (**59**), and (−)-fatouain F (**60**), along with two new bis-prenylcoumarins, (+)-fatouain G (**58**), and (+)-fatouain H (**59**), have been isolated from the whole plants of *Fatoua pilosa* [84]. (See Scheme 19.)

**Scheme 20:**
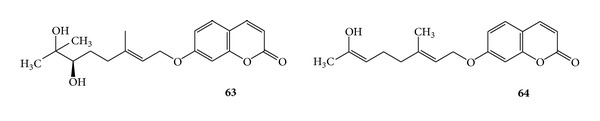


Marmin (**63**) is isolated from the bark. Imperatorin (**2**) and aurapten (**64**) are isolated from the fruit of *Aegle marmelos* (linn) Correa commonly known as Bael (or Bel) belonging to the family Rutaceae [29]. (See Scheme 20.)

## 5. Analysis of Coumarins by Different Methods

Various methods for the isolation and analysis of coumarins are chromatography (paper chromatography, thin layer chromatography, gas chromatography, and high-performance liquid chromatography), titrimetric and spectrophotometric (colorimetric and polarographic) methods. Methods for the analysis of coumarin derivatives stipulated by official pharmacopoeias (US Pharmacopoeia (23rd Edition), European Pharmacopoeia (3rd Edition, Suppl. 2001), and British Pharmacopoeia (16th Edition, 1998) and methods for coumarin determination in yellow sweet clover have been reviewed [85]. 

## 6. Conclusion

This paper covers natural coumarin lead compounds and their broad pharmacological properties and their methods of identification according to their official pharmacopoeias. Natural coumarins are of great interest due to their widespread pharmacological properties, and this attracts many medicinal chemists for further backbone derivatization and screening them as several novel therapeutic agents.

## Figures and Tables

**Table 1:** Different coumarin types and their pharmacological properties.

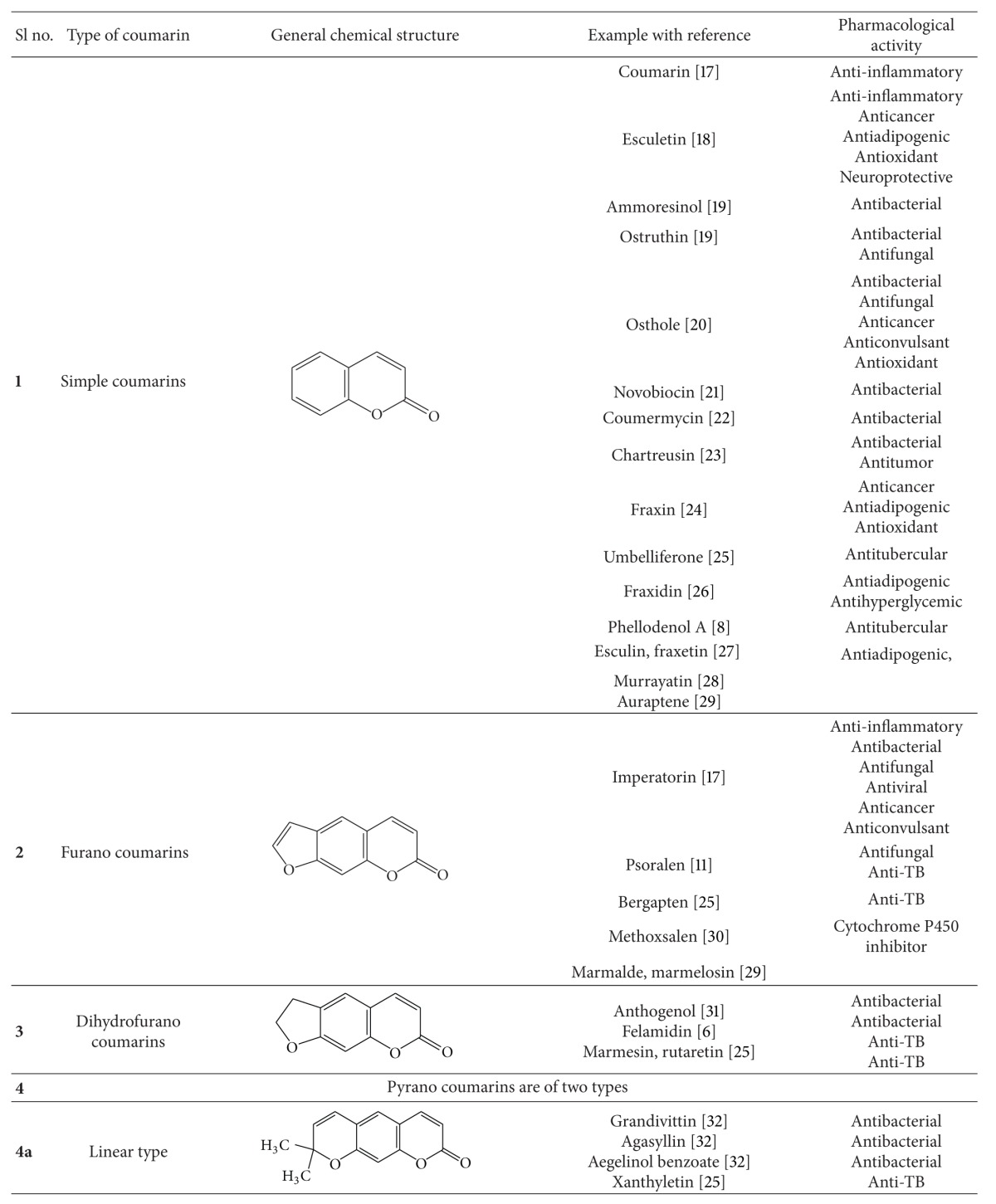 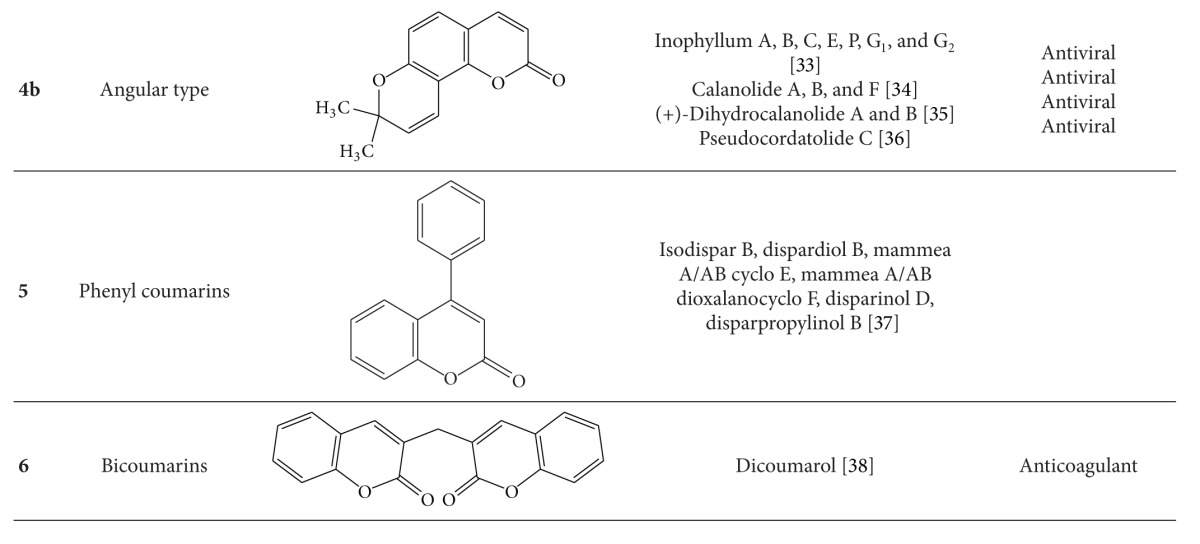

## References

[1] Aoyama Y, Katayama T, Yamamoto M, Tanaka H, Kon K (1992). A new antitumor antibiotic product, demethylchartreusin. Isolation and biological activities. *The Journal of Antibiotics*.

[2] Iranshahi M, Askari M, Sahebkar A, Hadjipavlou-Litina D (2009). Evaluation of antioxidant, anti-inflammatory and lipoxygenase inhibitory activities of the prenylated coumarin umbelliprenin. *DARU*.

[3] Evans WC (2009). *Trease and Evans Pharmacognosy*.

[4] Mead JA, Smith JN, Williams RT (1958). Studies in detoxication. 72. The metabolism of coumarin and of o-coumaric acid. *The Biochemical Journal*.

[5] Spino C, Dodier M, Sotheeswaran S (1998). Anti-HIV coumarins from calophyllum seed oil. *Bioorganic and Medicinal Chemistry Letters*.

[6] Rosselli S, Maggio AM, Faraone N (2009). The cytotoxic properties of natural coumarins isolated from roots of *Ferulago campestris* (Apiaceae) and of synthetic ester derivatives of aegelinol. *Natural Product Communications*.

[7] Atta-ur-Rahman, Shabbir M, Ziauddin Sultani S, Jabbar A, Choudhary MI (1997). Cinnamates and coumarins from the leaves of *Murraya paniculata*. *Phytochemistry*.

[8] Cohen AJ (1979). Critical review of the toxicology of coumarin with special reference to interspecies differences in metabolism and hepatotoxic response and their significance to man. *Food and Cosmetics Toxicology*.

[9] Fuller RW, Bokesch HR, Gustafson KR (1994). HIV-Inhibitory coumarins from latex of the tropical rainforest tree *Calophyllum teysmannii* var. inophylloide. *Bioorganic and Medicinal Chemistry Letters*.

[10] Choi J, Lee KT, Ka H, Jung WT, Jung HJ, Park HJ (2001). Constituents of the essential oil of the *Cinnamomum cassia* stem bark and the biological properties. *Archives of Pharmacal Research*.

[11] Bourgaud F, Hehn A, Larbat R (2006). Biosynthesis of coumarins in plants: a major pathway still to be unravelled for cytochrome P450 enzymes. *Phytochemistry Reviews*.

[12] Bogdal D (1998). Coumarins: fast synthesis by Knoevenagel condensation under microwave irradiation. *Journal of Chemical Research, Synopses*.

[13] Lake BG (1999). Coumarin metabolism, toxicity and carcinogenicity: relevance for human risk assessment. *Food and Chemical Toxicology*.

[14] Egan D, O’Kennedy R, Moran E, Cox D, Prosser E, Thornes RD (1990). The pharmacology, metabolism, analysis, and applications of coumarin and coumarin-related compounds. *Drug Metabolism Reviews*.

[15] Marshall ME, Mohler JL, Edmonds K (1994). An updated review of the clinical development of coumarin (1,2-benzopyrone) and 7-hydroxycoumarin. *Journal of Cancer Research and Clinical Oncology*.

[16] Murray RDH (1997). Naturally occuring plant coumarins. *Progress in the Chemistry of Organic Natural Products*.

[17] Piller NB (1975). A comparison of the effectiveness of some anti inflammatory drugs on thermal oedema. *British Journal of Experimental Pathology*.

[18] Witaicenis A, Seito LN, Di Stasi LC (2010). Intestinal anti-inflammatory activity of esculetin and 4-methylesculetin in the trinitrobenzenesulphonic acid model of rat colitis. *Chemico-Biological Interactions*.

[19] Hodák K, Jakesová V, Dadák V (1967). On the antibiotic effects of natural coumarins. VI. The relation of structure to the antibacterial effects of some natural coumarins and the neutralization of such effects. *Cesko-Slovenska Farmacie*.

[20] Wang CM, Zhou W, Li CX, Chen H, Shi ZQ, Fan YJ (2009). Efficacy of osthol, a potent coumarin compound, in controlling powdery mildew caused by *Sphaerotheca fuliginea*. *Journal of Asian Natural Products Research*.

[21] Chain EB (1958). Chemistry and biochemistry of antibiotics. *Annual Review of Biochemistry*.

[22] Gellert M, O’Dea MH, Itoh T, Tomizawa JI (1976). Novobiocin and coumermycin inhibit DNA supercoiling catalyzed by DNA gyrase. *Proceedings of the National Academy of Sciences of the United States of America*.

[23] Portugal J (2003). Chartreusin, elsamicin A and related anti-cancer antibiotics. *Current Medicinal Chemistry. Anti-Cancer Agents*.

[24] Whang WK, Park HS, Ham I (2005). Natural compounds, fraxin and chemicals structurally related to fraxin protect cells from oxidative stress. *Experimental and Molecular Medicine*.

[25] Chiang CC, Cheng MJ, Peng CF, Huang HY, Chen IS (2010). A novel dimeric coumarin analog and antimycobacterial constituents from *Fatoua pilosa*. *Chemistry and Biodiversity*.

[26] Yusupov MI, Sidyakin GP (1975). Fraxidin and isofraxidin from *Artemisia scotina*. *Chemistry of Natural Compounds*.

[27] Shin E, Choi KM, Yoo HS, Lee CK, Hwang BY, Lee MK (2010). Inhibitory effects of coumarins from the stem barks of *Fraxinus rhynchophylla* on adipocyte differentiation in 3T3-L1 cells. *Biological and Pharmaceutical Bulletin*.

[28] Barik BR, Dey AK, Chatterjee A (1983). Murrayatin, a coumarin from *Murraya exotica*. *Phytochemistry*.

[29] Farooq S (2005). *555 Medicinal Plants. Field and Laboratory Manual*.

[30] Kharasch ED, Hankins DC, Taraday JK (2000). Single-dose methoxsalen effects on human cytochrome P-450 2A6 activity. *Drug Metabolism and Disposition*.

[31] Chakthong S, Weaaryee P, Puangphet P (2012). Alkaloid and coumarins from the green fruits of *Aegle marmelos*. *Phytochemistry*.

[32] Basile A, Sorbo S, Spadaro V (2009). Antimicrobial and antioxidant activities of coumarins from the roots of *Ferulago campestris* (apiaceae). *Molecules*.

[33] Patil AD, Freyer AJ, Eggleston DS (1993). The inophyllums, novel inhibitors of HIV-1 reverse transcriptase isolated from the Malaysian tree, *Calophyllum inophyllum* Linn. *Journal of Medicinal Chemistry*.

[34] Kashman Y, Gustafson KR, Fuller RW (1992). The calanolides, a novel HIV-inhibitory class of coumarin derivatives from the tropical rainforest tree, *Calophyllum lanigerum*. *Journal of Medicinal Chemistry*.

[35] Newman RA, Chen W, Madden TL (1998). Pharmaceutical properties of related calanolide compounds with activity against human immunodeficiency virus. *Journal of Pharmaceutical Sciences*.

[36] McKee TC, Fuller RW, Covington CD (1996). New pyranocoumarins isolated from *Calophyllum lanigerum* and *Calophyllum teysmannii*. *Journal of Natural Products*.

[37] Crombie L, Games DE, McCormick A (1966). Isolation and structure of mammea A/BA, A/AB and A/BB: a group of 4-aryl-coumarin extractives of *Mammea americana* L. *Tetrahedron Letters*.

[38] Poole SK, Poole CF (1994). Thin-layer chromatographic method for the determination of the principal polar aromatic flavour compounds of the cinnamons of commerce. *The Analyst*.

[39] Huang GJ, Deng JS, Liao JC (2012). Inducible nitric oxide synthase and cyclooxygenase-2 participate in anti-inflammatory activity of imperatorin from *Glehnia littoralis*. *Journal of Agricultural and Food Chemistry*.

[40] Nadkarni A (1976). *Nadkarni's Indian Materia Medica*.

[41] Chang WS, Chang YH, Lu FJ, Chiang HC (1994). Inhibitory effects of phenolics on xanthine oxidase. *Anticancer Research*.

[42] Kwon OS, Choi JS, Islam MN (2011). Inhibition of 5-lipoxygenase and skin inflammation by the aerial parts of *Artemisia capillaris* and its constituents. *Archives of Pharmacal Research*.

[43] Fylaktakidou KC, Hadjipavlou-Litina DJ, Litinas KE, Nicolaides DN (2004). Natural and synthetic coumarin derivatives with anti-inflammatory/antioxidant activities. *Current Pharmaceutical Design*.

[44] Hirsh J, Dalen JE, Anderson DR (2001). Oral anticoagulants: mechanism of action, clinical effectiveness, and optimal therapeutic range. *Chest*.

[45] Nelsestuen GL, Zytkovicz TH, Howard JB (1974). The mode of action of vitamin K. Identification of *γ* carboxyglutamic acid as a component of prothrombin. *The Journal of Biological Chemistry*.

[46] Stenflo J, Fernlund P, Egan W, Roepstorff P (1974). Vitamin K dependent modifications of glutamic acid residues in prothrombin. *Proceedings of the National Academy of Sciences of the United States of America*.

[47] Whitlon DS, Sadowski JA, Suttie JW (1978). Mechanism of coumarin action: significance of vitamin K epoxide reductase inhibition. *Biochemistry*.

[48] Trivedi LS, Rhee M, Galivan JH, Fasco MJ (1988). Normal and warfarin-resistant rat hepatocyte metabolism of vitamin K 2,3-epoxide: evidence for multiple pathways of hydroxyvitamin K formation. *Archives of Biochemistry and Biophysics*.

[49] Fasco MJ, Hildebrandt EF, Suttie JW (1982). Evidence that warfarin anticoagulant action involves two distinct reductase activities. *The Journal of Biological Chemistry*.

[50] Choonara IA, Malia RG, Haynes BP (1988). The relationship between inhibition of vitamin K1 2,3-epoxide reductase and reduction of clotting factor activity with warfarin. *British Journal of Clinical Pharmacology*.

[51] Friedman PA, Rosenberg RD, Hauschka PV, Fitz-James A (1977). A spectrum of partially carboxylated prothrombins in the plasmas of coumarin-treated patients. *Biochimica et Biophysica Acta*.

[52] Malhotra OP, Nesheim ME, Mann KG (1985). The kinetics of activation of normal and *γ*-carboxyglutamic acid-deficient prothrombins. *The Journal of Biological Chemistry*.

[53] Nelsestuen GL (1976). Role of *γ* carboxyglutamic acid. An unusual protein transition required for the calcium dependent binding of prothrombin to phospholipid. *The Journal of Biological Chemistry*.

[54] Prendergast FG, Mann KG (1977). Differentiation of metal ion induced transitions of prothrombin fragment 1. *The Journal of Biological Chemistry*.

[55] Borowski M, Furie BC, Bauminger S, Furie B (1986). Prothrombin requires two sequential metal-dependent conformational transitions to bind phospholipid. Conformation-specific antibodies directed against the phospholipid-binding site on prothrombin. *The Journal of Biological Chemistry*.

[56] Baek NI, Ahn EM, Kim HY, Park YD (2000). Furanocoumarins from the root of *Angelica dahurica*. *Archives of Pharmacal Research*.

[57] Raja SB, Murali MR, Roopa K (2011). Imperatorin a furocoumarin inhibits periplasmic Cu-Zn SOD of Shigella dysenteriae their by modulates its resistance towards phagocytosis during host pathogen interaction. *Biomedicine & Pharmacotherapy*.

[58] Hestrin S, Feingold DS, Avigad G (1955). Synthesis of sucrose and other *β*-D-fructofuranosyl aldosides by levansucrase. *Journal of the American Chemical Society*.

[59] Teng CM, Lin CH, Ko FN, Wu TS, Huang TF (1994). The relaxant action of osthole isolated from *Angelica pubescens* in guinea-pig trachea. *Naunyn-Schmiedeberg’s Archives of Pharmacology*.

[60] Chou SY, Hsu CS, Wang KT, Wang MC, Wang CC (2007). Antitumor effects of osthol from *Cnidium monnieri*: an *in vitro* and *in vivo* study. *Phytotherapy Research*.

[61] Cisowski W, Sawicka U, Mardarowicz M, Asztemborska M, Luczkiewicz M (2001). Essential oil from herb and rhizome of *Peucedanum ostruthium* (L. Koch.) ex DC. *Zeitschrift für Naturforschung C*.

[62] Stout GH, Stevens KL (1964). The structure of costatolide. *Journal of Organic Chemistry*.

[63] Sancho R, Márquez N, Gómez-Gonzalo M (2004). Imperatorin inhibits HIV-1 replication through an Sp1-dependent pathway. *The Journal of Biological Chemistry*.

[64] Luo KW, Sun JG, Chan JY (2011). Anticancer effects of imperatorin isolated from *Angelica dahurica*: induction of apoptosis in HepG2 cells through both death-receptor and mitochondria-mediated pathways. *Chemotherapy*.

[65] Yang D, Gu T, Wang T, Tang Q, Ma C (2010). Effects of osthole on migration and invasion in breast cancer cells. *Bioscience, Biotechnology and Biochemistry*.

[66] Yun ES, Park SS, Shin HC (2011). p38 MAPK activation is required for esculetin-induced inhibition of vascular smooth muscle cells proliferation. *Toxicology in Vitro*.

[67] Lee CR, Shin EJ, Kim HC (2011). Esculetin inhibits N-methyl-D-aspartate neurotoxicity via glutathione preservation in primary cortical cultures. *Laboratory Animal Research*.

[68] Crichton EG, Waterman PG (1978). Dihydromammea C/OB: a new coumarin from the seed of *Mammea africana*. *Phytochemistry*.

[69] Schwalbe CH, Waterman PG (1983). Structure of 5, 7-dihydroxy-8-(2-methylbutyryl)-4-n-pentyl-3, 4-dihydrocoumarin (dihydromammea C/OB), C_19_H_26_O_5_. *Acta Crystallographica Section C*.

[70] Nguelefack-Mbuyo PE, Nguelefack TB, Dongmo AB (2008). Anti-hypertensive effects of the methanol/methylene chloride stem bark extract of *Mammea africana* in l-NAME-induced hypertensive rats. *Journal of Ethnopharmacology*.

[71] Tchamadeu MC, Dzeufiet PDD, Nouga CCK (2010). Hypoglycaemic effects of *Mammea africana* (Guttiferae) in diabetic rats. *Journal of Ethnopharmacology*.

[72] Namba T, Morita O, Huang SL, Goshima K, Hattori M, Kakiuchi N (1988). Studies on cardio-active crude drugs; I. Effect of coumarins on cultured myocardial cells. *Planta Medica*.

[73] Gantimur D, Syrchina AI, Semenov AA (1986). Khellactone derivatives from *Phlojodicarpus sibiricus*. *Chemistry of Natural Compounds*.

[74] Luszczki JJ, Wojda E, Andres-Mach M (2009). Anticonvulsant and acute neurotoxic effects of imperatorin, osthole and valproate in the maximal electroshock seizure and chimney tests in mice: a comparative study. *Epilepsy Research*.

[75] Chen X, Pi R, Zou Y (2010). Attenuation of experimental autoimmune encephalomyelitis in C57 BL/6 mice by osthole, a natural coumarin. *European Journal of Pharmacology*.

[76] Tinel M, Belghiti J, Descatoire V (1987). Inactivation of human liver cytochrome P-450 by the drug methoxsalen and other psoralen derivatives. *Biochemical Pharmacology*.

[77] Kim NY, Pae HO, Ko YS (1999). In vitro inducible nitric oxide synthesis inhibitory active constituents from *Fraxinus rhynchophylla*. *Planta Medica*.

[78] Fort DM, Rao K, Jolad SD, Luo J, Carlson TJ, King SR (2000). Antihyperglycemic activity of *Teramnus labialis* (Fabaceae). *Phytomedicine*.

[79] Kim SH, Kang KA, Zhang R (2008). Protective effect of esculetin against oxidative stress-induced cell damage via scavenging reactive oxygen species. *Acta Pharmacologica Sinica*.

[80] Hirsch AM, Longeon A, Guyot M (2002). Fraxin and esculin: two coumarins specific to *Actinidia chinensis* and *A. deliciosa* (kiwifruit). *Biochemical Systematics and Ecology*.

[81] Wang C, Pei A, Chen J (2012). A natural coumarin derivative esculetin offers neuroprotection on cerebral ischemia/reperfusion injury in mice. *Journal of Neurochemistry*.

[82] Guilet D, Séraphin D, Rondeau D, Richomme P, Bruneton J (2001). Cytotoxic coumarins from *Calophyllum dispar*. *Phytochemistry*.

[83] Crombie L, Games DE, Haskins NJ, Reed GF (1972). Extractives of *Mammea americana* L—part III: Identification of new coumarin relatives of mammea B/BA, B/BB, and B/BC having 5,6-annulation and higher oxidation levels. *Journal of the Chemical Society, Perkin Transactions 1*.

[84] Chiang CC, Cheng MJ, Huang HY, Chang HS, Wang CJ, Chen IS (2010). Prenyl coumarins from *Fatoua pilosa*. *Journal of Natural Products*.

[85] Lozhkin AV, Sakanyan EI (2006). Natural coumarins: methods of isolation and analysis. *Pharmaceutical Chemistry Journal*.

